# Supplementary material for: Analysis of the Spatiotemporal Development of Hematopoietic Stem and Progenitor Cells in the Early Human Embryo
Source: Stem Cell Reports. 2019 Apr 4;12(5):1056–68. doi: 10.1016/j.stemcr.2019.03.003 (PMC6525107; doi:10.1016/j.stemcr.2019.03.003)
Supplement: Document S1. Figures S1–S4 and Table S1 [file mmc1.pdf]

Stem Cell Reports, Volume *12*

Supplemental Information

Analysis of the Spatiotemporal Development of Hematopoietic Stem and Progenitor Cells in the Early Human Embryo

Jennifer Easterbrook, Stanislav Rybtsov, Sabrina Gordon-Keylock, Andrejs Ivanovs, Samir Taoudi, Richard A. Anderson, and Alexander Medvinsky

Table S1. Analysis of long-term repopulation by cultured human embryonic tissue (non-AGM). Relates to Table 1.

Experiment	CS	Tissue	Conditions	e.e./ mouse	no. engrafted/no. injected
A	15	UC	SCF, TGFβi, ROCKi	0.4	0/2
B	16	UC	SCF	0.25	0/4
C	17	EL	SCF, TGFβi, ROCKi	0.45	0+2[A]/2
		UC		0.45	0/2
E	13	UC	SCF	0.5	0/2
		VA		0.5	0/2
		YS		0.5	0/2
F	16	UC	SCF	0.5	0/2
		VA		0.5	0/2
		YS		0.5	0/2
H	16	EL	SCF	0.4	0+1[A]/1
I	17	EL	SCF	0.5	**1**/1
J	13	YS	SCF, FLT3L	0.3	0/3

Long-term multilineage haematopoietic repopulation (HSCs)
[A]Low level (<1%) engraftment in bone marrow.

The number of repopulated NSG mice relative to the number of mice injected and analysed are shown. The embryo equivalent (e.e.) received by each mouse refers to the e.e. of each *part* of the AGM region. Experiment letters correspond to experiments in Table 1. SCF is 100ng/ml unless stated as 300 (ng/ml), TGFβ inhibitor 5μM, ROCK inhibitor 10μM, FLT3L 100ng/ml. CS, Carnegie stage. TGFβi, TGFβ inhibitor. ROCKi, ROCK inhibitor. YS, yolk sac. UC, umbilical cord. VA, vitelline artery. EL, embryonic liver.

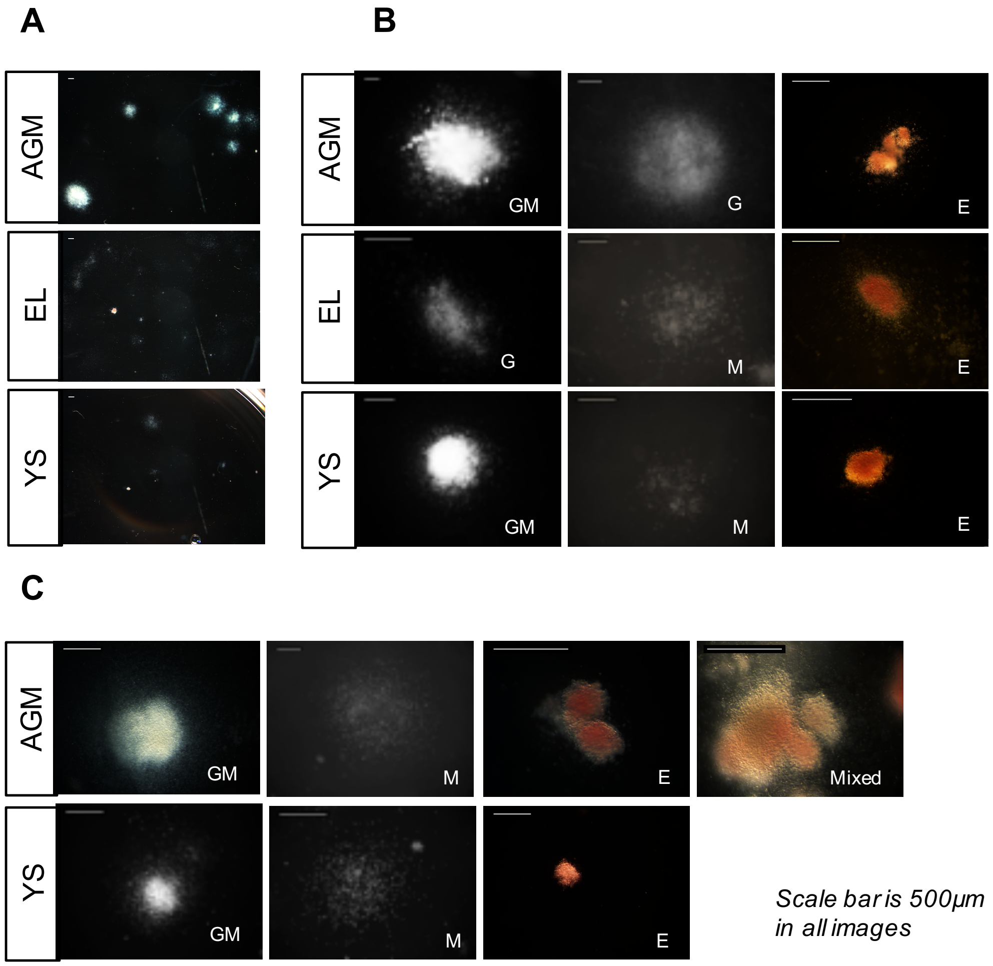

Figure S1. **CFU-Cs from human embryonic tissue before and after explant culture. Relates to Figure 1 and Figure 2B.**
Representative images of CFU-Cs from the AGM, embryonic liver (EL), and yolk sac (YS) before culture (A & B) and after 7 days of explant culture with SCF 100ng/ml (C). Images for all tissues were taken 10 days after cells were seeded in methylcellulose at appropriate embryo equivalents to allow accurate counting after culture. All scale bars represent 500µm.
A: CFU-Cs from a fresh (not cultured) CS16 embryo. These lower power images were all taken at the same magnification and show the different sizes of colonies from different tissues.
B: Example images of different colony types: Myeloid (granulocyte (G), macrophage (M), granulocyte/macrophage (GM)) and erythroid colonies are depicted for each tissue. No mixed colonies were produced from this embryo before explant culture.
C: CFU-Cs from a CS14 embryo after explant culture showing granulocyte/macrophage (GM), macrophage (M) and erythroid (E) colonies produced from both AGM and YS. Mixed colonies were produced from the cultured AGM tissue only.

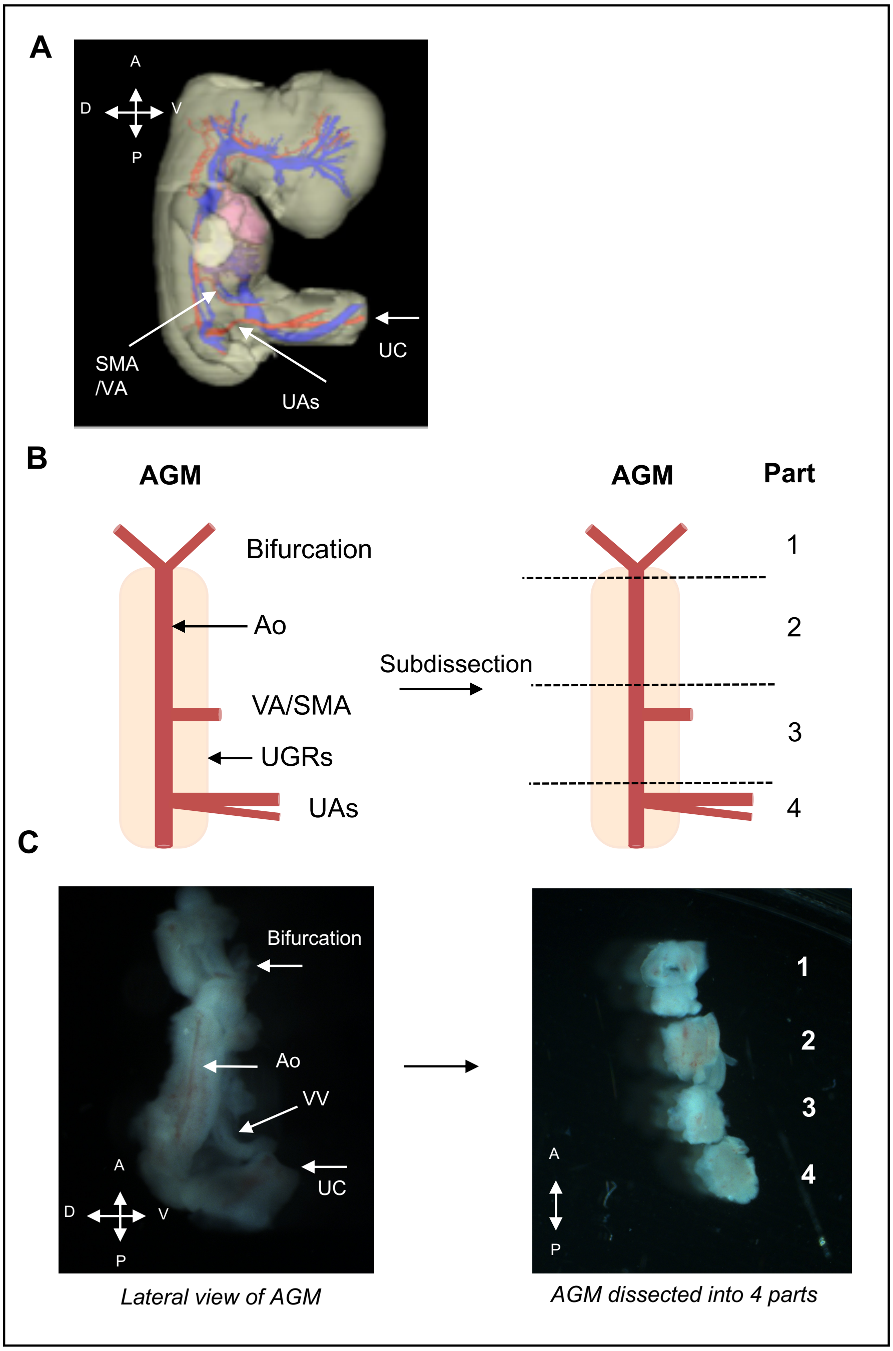

Figure S2. Sub-dissection of the human AGM along the rostro-caudal axis. Relates to Figure 3.

A: Circulatory system of a CS17 human embryo. Arteries (red) and veins (blue). The superior mesenteric artery (SMA), a derivative of the vitelline artery (VA) and umbilical arteries (UAs) within the umbilical cord (UC) are marked. Image adapted from *'The Virtual Human Embryo', hosted by EHD* (Gasser & Cork).

B: Schematic of sub-dissection to divide AGM into 4 regions along the rostro-caudal axis, performed for all CS13-17 embryos. Part 1: From the rostral part of the urogenital ridges (UGRs) including the aortic bifurcation

Part 2: From above the VA/ SMA to the rostral part of the UGRs

Part 3: From above the UAs entry points including the VA/ SMA

Part 4: Included the UAs entry points and the aorta below

C: Example of a CS17 embryo used in the experiments showing the lateral view of the AGM and the 4 parts after dissection.

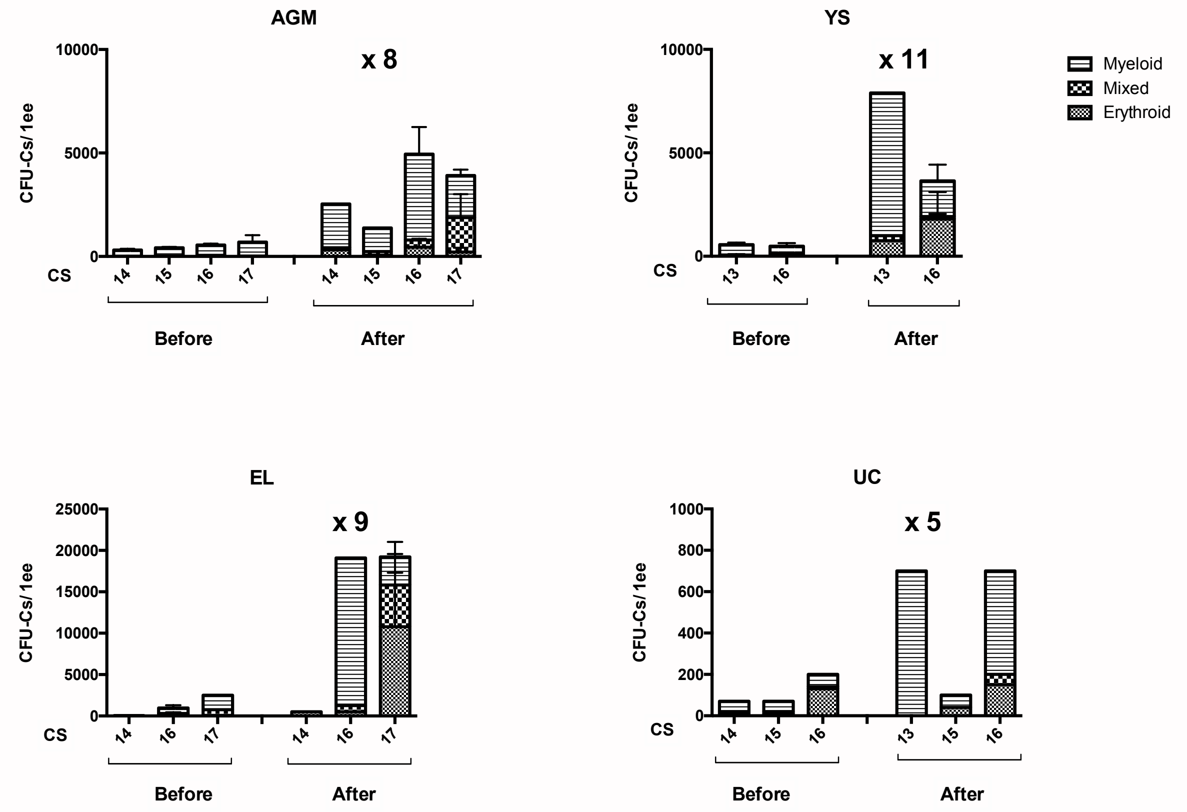

Figure S3. **Explant culture can expand committed haematopoietic progenitors from human embryonic tissue . Relates to Figure 2.**

Charts show the number and type (myeloid, mixed or erythroid) of CFU-Cs in the human AGM (A), yolk sac (YS) (B), embryonic liver (EL) (C), and umbilical cord (UC) (D) before and after explant culture for 7 days. Tissue was dissected and cultured at the air-liquid interface in standard embryo culture medium with addition of SCF 100ng/ml. The mean fold change in CFU-C numbers after culture is shown above the graph. Number of CFU-Cs before culture are the mean of separate experiments shown in Figure 1. n=1 except where error bars are given where n=2 and for AGM CS16 n=3. ee, embryo equivalent.

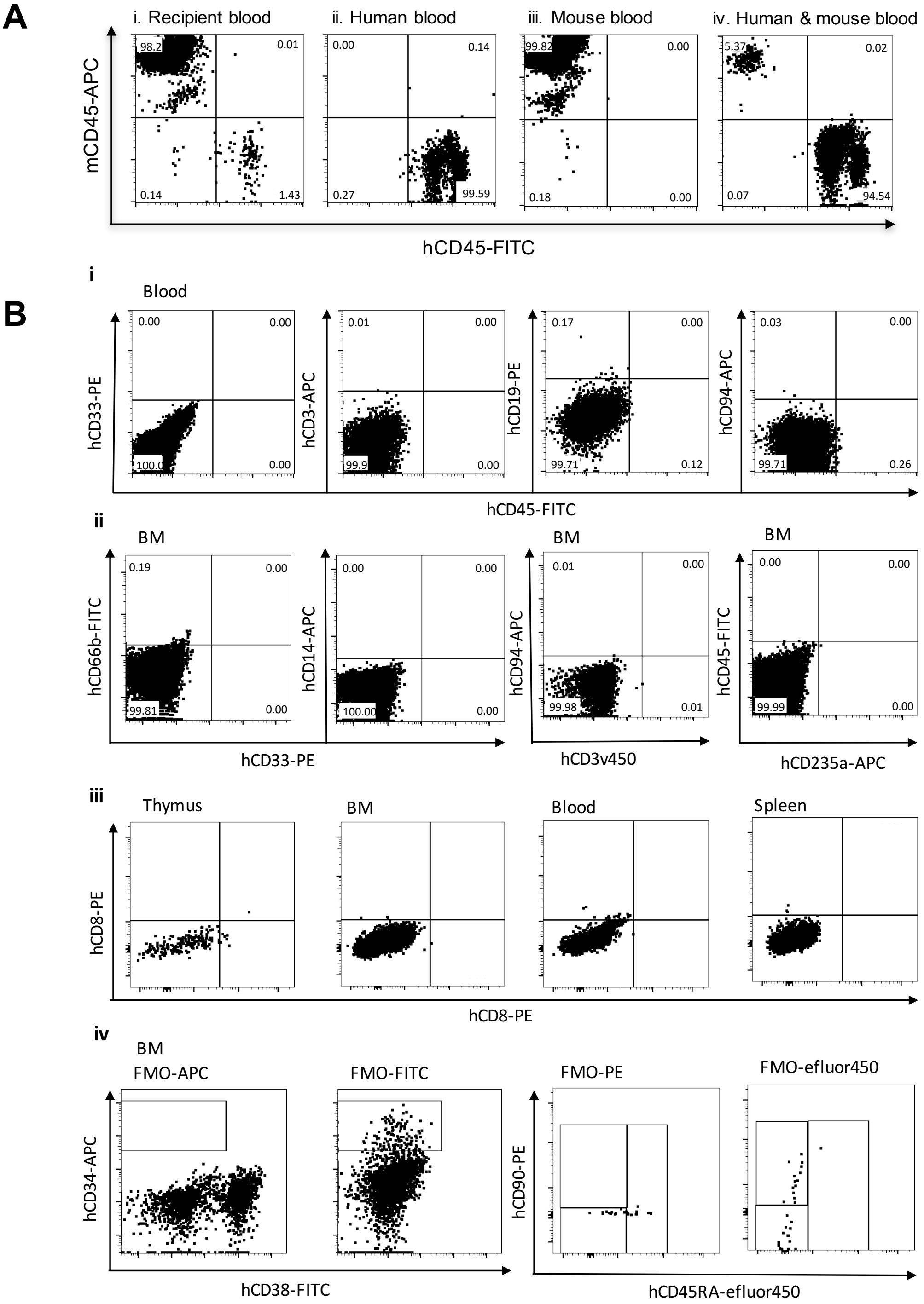

Figure S4. **Flow cytometry controls for multilineage analysis of haematopoietic repopulation. Relates to Figure 4.**

A: Representative plots showing analysis of peripheral blood of recipient NSG mice by flow cytometry. i. Engrafted recipient mouse blood from experiment G at 4 months, ii. Human peripheral blood (positive flow cytometry control), iii. Non-injected NSG mouse blood (negative control), iv. Human blood spiked with mouse blood, a control commonly used; plot iv was taken from a separate experiment from i-iii for illustration purposes.

B: Representative flow cytometry plots showing controls for gating human myeloid, T, B, NK cells (i), granulocytes, macrophages, NK/NKT cells and erythroid cells (ii), CD8/CD4 T cells (iii) and haematopoietic stem and progenitor cells (iv) in blood, bone marrow, spleen and thymus of primary recipient mice. Cells were generally gated using cells taken from non-engrafted primary recipient mice which had been sub-lethally irradiated & initially injected with PBS. This allowed gates to be drawn based on the autofluorescence of cells. Fluorescence minus one (FMO) controls were also used for each experiment and the gates drawn here were checked against each FMO control. Isotype controls were not used having carefully considered their advantages and disadvantages (Keeney *et al*, 1998; O'Gorman & Thomas, 1999).
