# Supplementary material for: Analysis of the Spatiotemporal Development of Hematopoietic Stem and Progenitor Cells in the Early Human Embryo
Source: Stem Cell Reports. 2019 Apr 4;12(5):1056–68. doi: 10.1016/j.stemcr.2019.03.003 (PMC6525107; doi:10.1016/j.stemcr.2019.03.003)
Supplement: Document S2. Article plus Supplemental Information [file mmc2.pdf]

Stem Cell Reports
Article
OPEN ACCESS

Analysis of the Spatiotemporal Development of Hematopoietic Stem and Progenitor Cells in the Early Human Embryo

Jennifer Easterbrook,[1] Stanislav Rybtsov,[1] Sabrina Gordon-Keylock,[1] Andrejs Ivanovs,[1,2] Samir Taoudi,[3,4,5] Richard A. Anderson,[6] and Alexander Medvinsky[1,*]

[1]Ontogeny of Haematopoietic Stem Cells Group, Institute for Stem Cell Research, MRC Centre for Regenerative Medicine, University of Edinburgh, Edinburgh EH16 4UU, UK
[2]Institute of Anatomy and Anthropology, Riga Stradiņš University, Riga 1007, Latvia
[3]Molecular Medicine Division, The Walter and Eliza Hall Institute of Medical Research, Melbourne, VIC 3052 Australia
[4]Department of Medical Biology, University of Melbourne, Melbourne, VIC 3052, Australia
[5]Cancer and Haematology Division, The Walter and Eliza Hall Institute of Medical Research, Melbourne, VIC 3052, Australia
[6]MRC Centre for Reproductive Health, The Queen's Medical Research Institute, University of Edinburgh, Edinburgh EH16 4TJ, UK
*Correspondence: a.medvinsky@ed.ac.uk
https://doi.org/10.1016/j.stemcr.2019.03.003

SUMMARY

Definitive hematopoietic stem cells (HSCs) first emerge in the aorta-gonad-mesonephros (AGM) region in both mice and humans. An *ex vivo* culture approach has enabled recapitulation and analysis of murine HSC development. Knowledge of early human HSC development is hampered by scarcity of tissue: analysis of both CFU-C and HSC development in the human embryo is limited. Here, we characterized the spatial distribution and temporal kinetics of CFU-C development within early human embryonic tissues. We then sought to adapt the murine *ex vivo* culture system to recapitulate human HSC development. We show robust expansion of CFU-Cs and maintenance, but no significant expansion, of human HSCs in culture. Furthermore, we demonstrate that HSCs emerge predominantly in the middle section of the dorsal aorta in our culture system. We conclude that there are important differences between early mouse and human hematopoiesis, which currently hinder the quest to recapitulate human HSC development *ex vivo*.

INTRODUCTION

Hematopoietic stem cells (HSCs) develop in the aorta-gonad-mesonephros (AGM) region, marking the onset of life-long adult hematopoiesis (Ivanovs et al., 2011; Medvinsky and Dzierzak, 1996; Müller et al., 1994). Various lines of evidence indicate that in both mice and humans, HSCs mature within intra-aortic hematopoietic clusters (IAHCs), budding predominantly from the ventral floor of the dorsal aorta, and co-express endothelial and hematopoietic markers: CD34, CD144 (VE-cadherin), CD45, RUNX1 (Ivanovs et al., 2014; Souilhol et al., 2016; Tavian et al., 1996, 1999; Taylor et al., 2010; Yokomizo and Dzierzak, 2010). In mouse and humans, definitive HSCs, detectable by direct transplantation into adult recipients, emerge in the AGM region in low numbers (~1 HSC) (Ivanovs et al., 2011; Kumaravelu et al., 2002; Rybtsov et al., 2016).

Knowledge of the process of HSC maturation and expansion will be essential for directing the successful development of HSCs *in vitro*. Due to the rarity of suitable embryonic material, analysis of early HSC development in humans has significantly lagged behind mouse studies. In the mouse, an AGM *ex vivo* culture system facilitates recapitulation of HSC development, enables the analysis of the developing HSC hierarchy and identification of HSC molecular regulators (Rybtsov et al., 2011, 2014; Souilhol et al., 2016; Taoudi et al., 2008). This culture system, functionally validated by long-term repopulation transplantation assays, has demonstrated a concealed dramatic expansion of immature HSC precursors (pre-HSCs) that occurs in the mouse AGM region prior to colonization of the fetal liver (Rybtsov et al., 2016). Evidence of human precursors of HSCs has not yet been sought. We hypothesized that pre-HSCs also exist in the human AGM and that their existence could be established using similar culture conditions to those employed for the maturation of mouse HSCs.

Understanding the precise location of HSC emergence will also be fundamental for future investigation of human embryonic hematopoiesis and ultimately achieving the generation of HSCs *in vitro*. Earlier studies in the human embryo demonstrated that IAHCs, composed of hematopoietic $CD34^+CD45^+$ cells, are found exclusively in the ventral side of the dorsal aorta (AoV), emerging at Carnegie stage (CS) 12–13 (27 days post conception [dpc]) and disappearing by CS17 (39–42 dpc) (Tavian et al., 1996, 1999). This exclusive location of clusters in AoV correlates with the detection of HSCs only in AoV in the human embryo (Ivanovs et al., 2011). We hypothesized that the location of HSCs would also correlate with the distribution of hematopoietic progenitor cells in the AGM region. Basic analysis of committed human myeloid progenitor cells (CFU-Cs) in the human embryo

This is an open access article under the CC BY license (http://creativecommons.org/licenses/by/4.0/).

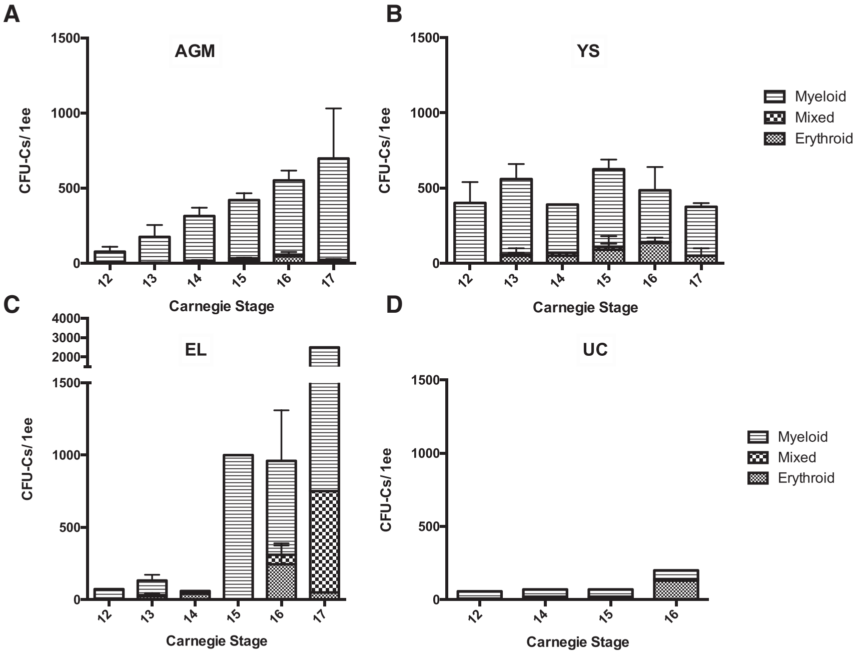

Figure 1. Spatiotemporal Distribution of Committed Hematopoietic Progenitors in the Human Embryo

The number and type of CFU-Cs at different Carnegie stages (CS) are shown for the AGM region (A), yolk sac (YS) (B), embryonic liver (EL) (C), and umbilical cord (UC) (D). For these experiments, embryonic tissue was staged, dissected, dissociated, and plated directly into methylcellulose and analyzed 10–14 days later. For AGM, n ≥ 4. For YS, n = 2 except CS14 where n = 1. For EL, n = 1 except where error bars are shown where n = 4 (CS13) and n = 2 (CS16). For UC, n = 1. Error bars show the standard error of the mean (SEM). ee, embryonic equivalent. See also Figure S1.

is currently limited (Huyhn et al., 1995; Tavian et al., 2001). Here, we aimed to advance our understanding of early human hematopoietic development by temporal and spatial characterization of CFU-Cs and HSCs generated during the period of embryonic development between CS14 and CS17, when definitive HSCs and IAHCs are detectable in the human AGM region (Ivanovs et al., 2011; Tavian et al., 1996, 1999). We also aimed to establish a human AGM culture system, similar to the mouse system, which would recapitulate the *in vivo* process of HSC maturation and potentially reveal the presence of human pre-HSCs. We focused on a major regulator of HSC development, stem cell factor (SCF, KIT ligand) expressed in the AGM region, which efficiently supports HSC maturation in mouse AGM explants (Rybtsov et al., 2014; Souilhol et al., 2016).

We present quantitative distribution and growth kinetics of CFU-C development across hematopoietic tissues. The analysis of the *ex vivo* culture of the human AGM regions showed robust CFU-C expansion, but minimal or no HSC maturation/expansion. Although we revealed that definitive HSCs emerge predominantly in the middle section of the dorsal aorta, the failure to establish a productive human AGM culture system prevents us currently from gaining insight into human pre-HSC development. We conclude that despite clear evolutionary conservation of hematopoietic development across vertebrate species, some important differences between mouse and human exist that currently hinder analysis of HSC development in the human AGM region.

RESULTS

Spatial and Temporal Distribution of Hematopoietic Progenitors in the Early Human Embryo

To establish the spatial and temporal distribution of hematopoietic progenitors during early human development, we performed CFU-C assays during and just before the developmental window for HSCs/IAHCs in the human AGM region (CS12–17). CFU-Cs in the AGM region showed a linear increase in total numbers from 75 ± 35 at CS12 to 700 ± 340 at CS17 (Figure 1). In the yolk sac, total CFU-C numbers remained more constant throughout this period (400 ± 140 at CS12 and 375 ± 75 at CS17). Meanwhile, in the embryonic liver, low CFU-C numbers (range, 40–210) were observed until CS14, followed by a dramatic rise at CS 15/16 (range, 700–1,100) with a further increase at CS17 (2,500). CFU-C numbers in the umbilical cord were low at all stages and fluctuated between 60 (at CS12) and 200 (at CS16). Notably, the size of myeloid colonies from the AGM region appeared larger than from other embryonic tissues (Figure S1).

With regard to colony morphology, CFU-Cs both in the AGM region and the yolk sac were predominantly granulocyte/macrophage (myeloid) colonies and a sizable fraction of relatively large erythroid colonies. A slight increase in erythroid colonies in the yolk sac occurred concurrently with their peak in the embryonic liver, AGM, and umbilical cord at CS16, potentially consistent with colonization of the embryo body with yolk sac progenitors. We also observed an increased complexity of colonies generated

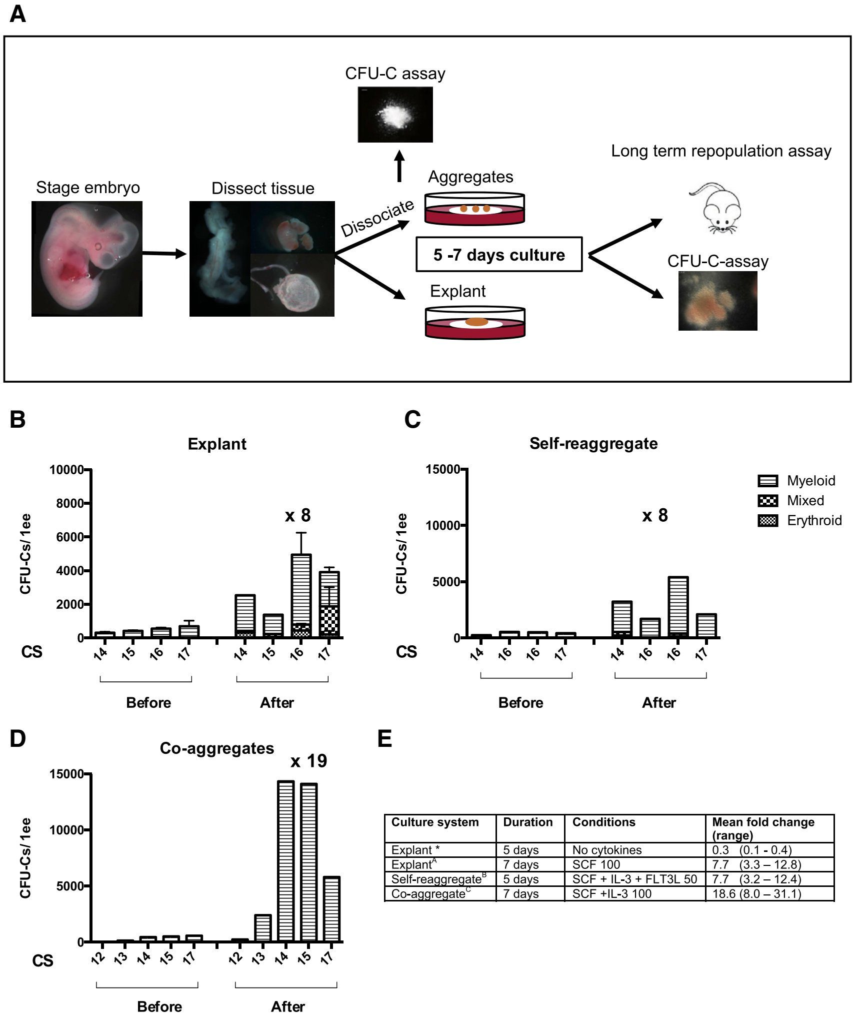

Culture system	Duration	Conditions	Mean fold change (range)
Explant *	5 days	No cytokines	0.3 (0.1 - 0.4)
Explant[A]	7 days	SCF 100	7.7 (3.3 – 12.8)
Self-reaggregate[B]	5 days	SCF + IL-3 + FLT3L 50	7.7 (3.2 – 12.4)
Co-aggregate[C]	7 days	SCF +IL-3 100	18.6 (8.0 – 31.1)

Figure 2. Testing *Ex Vivo* Culture Systems for Expansion of Hematopoietic Progenitors

(A) Human embryos were staged, dissected, and either dissociated to make aggregates or cultured as explants at the air-liquid interface. When aggregates were made, aliquots were taken for the CFU-C assay (typically 0.1 ee). Aggregates were either self-reaggregated or co-aggregated with OP9 stromal cells. All tissues were cultured for 5–7 days at the air-liquid interface with variable addition of cytokines/small molecules. After culture, the tissue was dissociated and injected into sub-lethally irradiated NSG mice. After culture of both aggregates and explants, aliquots were taken for the CFU-C assay (up to 0.1 ee). The images of dissected tissue depict the lateral view of an AGM (left), embryonic liver (top right), and yolk sac (bottom right).

(B–D) Number and type of CFU-Cs before and after culture using different culture systems; explant culture for 7 days (B), self-reaggregates cultured for 5 days (C), and co-aggregates with OP9s cultured for 7 days (D). SCF, IL3, FLT3LG 50–100 ng/mL were added as indicated in (E). Numbers shown above the graph indicate the mean fold expansion of the experiments shown. For (B), n = 3 for CS16, and n = 2 for CS17, and numbers before culture are taken from experiments with different embryos (see Figure 1). For (C) and (D), all bars are single experiments showing before and after culture from the same embryo. See also Figures S1 and S3.

(E) The mean fold increase after culture with different culture conditions corresponding to the charts above. *Indicates where the data are not shown on the above graphs. Here, three experiments only were performed with CS17 embryos. Error bars show SEM. ee, embryo equivalent. CS, Carnegie stage.

by livers from CS15 to CS17 with progressive appearance of mixed colonies (CFU-MIX) representing more immature progenitors (Figure 1). This was not observed in other tissues including the AGM region.

Establishing a Human AGM Explant Culture System

For the explant culture, AGM regions were dissected and cultured for 7 days in the presence of 100 ng/mL SCF (Figure 2), based on its efficient support of HSC maturation in the mouse AGM region (Rybtsov et al., 2014). We observed a 7.7-fold increase (range, 3.3–12.8) in CFU-C numbers across all CS13-17 AGM explants (n = 7). The number of CFU-Cs before culture was taken from other independent experiments since explants cannot be disrupted prior to culture (Figure 1A). We noted the appearance of CFU-MIX in cultured AGM regions that were not present before culture, suggesting their generation in explants. The macroscopic appearance of other types of colonies before and after culture was similar (Figure S1). Explants of other embryonic tissues (umbilical cord, yolk sac, liver) also showed expansion of CFU-Cs ranging from a mean fold change of 5 for the umbilical cord to 11 for the yolk sac across all experiments (Figure S3).

We next tested whether human HSCs could mature in the AGM culture. Between CS14 and CS17, the AGM region contains ~1 definitive HSC, which can provide long-term multilineage hematopoietic engraftment in NSG mice upon transplantation (Ivanovs et al., 2011). Initial independent experiments (n = 4) were performed using the whole AGM region or anterior and posterior halves (cut along the rostra-caudal axis) in the presence of exogenous SCF (Table 1; Experiments A–D). After 7 days of culture,

Table 1. Analysis of Long-Term Repopulation of NSG Mice by Cultured Human AGM

	CS	AGM Region	Conditions	Embryonic Equivalent/Mouse	No. Engrafted/No. Injected
**Initial Experiments**					
A	15	Anterior	SCF 300	0.14	0 + 3[a]/3
		Posterior	SCF	0.14	0 + 3[a]/3
B	16	Anterior	SCF	0.5	**1**/2
		Posterior		0.5	0/2
C	17	Anterior	SCF, TGF-βi, ROCKi	0.28	0 + 3[a]/3
		Posterior	SCF	0.28	**3**/3
D	17	Whole AGM	SCF, TGF-βi, ROCKi	0.15	**1**/6
**Mapping Experiments**					
E	13	Part 1	SCF	0.96	0/1
		Part 2		0.96	**1**[b]/1
		Part 3		0.96	0/1
		Part 4		0.96	0/1
F	16	Part 1	SCF	0.96	0/1
		Part 2		0.96	**1**/1
		Part 3		0.96	**1**/1
		Part 4		0.96	0/1
G	16	Part 1	SCF	0.3	0/2
		Part 2		0.3	**1** + 1[a]/2
		Part 3		0.3	**1**/3
		Part 4		0.3	0/3
H	16	Part 1	SCF	0.3	0/3
		Part 2		0.3	2[a]/3
		Part 3		0.3	0/2
		Part 4		0.3	0/3
I	17	Part 2	SCF	0.3	**1** + 1[a]/2
		Part 3		0.3	**1**/1
		Part 4		0.3	**1**/2

The number of repopulated NSG mice relative to the number of mice injected and analyzed are shown. For example, **1** + 1[a]/2 means one of the two mice injected had long-term multilineage repopulation at >1%; in the other mouse low-level engraftment was detected. Parts 1–4 are described in Figure S2 and experiments correspond to those in Tables 2 and 3. The embryo equivalent (ee) received by each mouse refers to the ee of each part of the AGM region. SCF is 100 ng/mL unless stated as 300 (ng/mL), TGF-β inhibitor 5 μM, ROCK inhibitor 10 μM, FLT3LG 100 ng/mL. CS, Carnegie stage. TGF-βi, TGF-β inhibitor. ROCKi, ROCK inhibitor.

[a]Low level (<1%) engraftment in bone marrow.

[b]Full multilineage analysis not performed.

AGM explants were dissociated and injected intravenously into irradiated NSG mice. Every 1–2 months post transplantation, the peripheral blood of recipient animals was assessed for donor-derived hematopoietic contribution using a combination of anti-mouse and anti-human CD45 antibodies. Multilineage engraftment was assessed

in blood, bone marrow, and spleen at least 3 months post transplantation. From a total of 22 recipients, five were reconstituted with >1% human CD45$^+$ cells and nine recipients had only low level (<1%) of bone marrow engraftment with no engraftment in peripheral blood. In one variant included in these experiments, one cultured posterior half of a CS17 AGM region showed >1% engraftment of three recipients, which is more than is expected before culture (Table 1, Experiment C). In two of the experiments with CS17 AGM regions, we used transforming growth factor β (TGF-β) inhibitor (5 mM) and ROCK inhibitor (10 mM) as these might improve the outcome, but these cultures showed no clear benefit compared with SCF only cultures (Table 1, Experiments C and D). Overall, the frequencies of repopulation showed that in contrast to mouse AGM culture systems, human AGM explants failed to support HSC maturation (expansion), but rather showed maintenance of solitary definitive HSCs pre-existing in fresh AGM region (Ivanovs et al., 2011). It is unclear whether one case of repopulation of three recipients by a single half of cultured CS17 AGM region (Table 1, Experiment C) was a result of unusually high contents of definitive HSCs (three or more) in the fresh tissue, or because in this particular case some HSC maturation occurred.

Explants of other embryonic tissues obtained from the same embryos (yolk sac, umbilical cord, and vitelline arteries) have not shown the presence of definitive HSCs after transplantation into NSG mice (cultures in the presence of SCF, TGF-β inhibitor, ROCK inhibitor, and/or FLT3 ligand [FLT3LG]) (Table S1). For cultured explants of embryonic livers, three of three NSG recipients had low-level bone marrow engraftment in two independent experiments (Table S1, Experiments C and H, CS16 and CS17), and in one independent experiment, long-term multilineage was observed (CS17) (Table S1, Experiment I), suggesting the possible beginning of liver colonization at this stage.

Hematopoietic Capacity of Self-Reaggregate and Co-aggregate Cultures

Self-reaggregate and co-aggregate cultures of the AGM region have been a powerful method for analysis of mechanisms underlying HSC development in the mouse AGM region (Rybtsov et al., 2011, 2014; Souilhol et al., 2016; Taoudi et al., 2008). Here, for the self-reaggregate cultures, single-cell suspensions of CS14–17 AGM regions were reaggregated (10^5 cells/reaggregate) and cultured in the presence of SCF, interleukin 3 (IL3), FLT3LG (50 ng/mL each) for 5 days. In contrast to explants, dissociation of cells enabled multiple aliquots to be prepared from one embryo and therefore direct comparison of CFU-C numbers in fresh tissues and after culture. Similar to explant cultures, CFU-C numbers increased 7.7-fold (range, 3.2–12.4) (Figure 2C).

For co-aggregate cultures, 10^5 AGM cells obtained from CS12–17 AGM regions were aggregated with 10^5 OP9 cells and cultured with SCF and IL3 (100 ng/mL each) for 7 days. We observed an 18.6-fold increase (range, 8.0–31.1) in CFU-C numbers after 7 days in co-aggregates across all experiments, which is higher than with both self-reaggregate and explant cultures (Figures 2D and 2E).

We then performed eight independent experiments where CS12–17 AGM regions were self-reaggregated and after being cultured for 7 days, were transplanted into irradiated NSG recipients. Only in one experiment were we able to detect long-term repopulation (data not shown). In a further six independent experiments, we performed co-aggregation of the CS12–17 AGM region with OP9s for 7 days in the presence of exogenous SCF and IL3 100 ng/mL. None of the experiments gave long-term hematopoietic reconstitution of NSG mice after transplantation (data not shown).

Definitive HSCs Are Localized Mainly to the Central Portion of the Human AGM Region

HSCs are known to emerge from the ventral side of the dorsal aorta (Ivanovs et al., 2014) but their more precise location along the rostro-caudal axis remains unknown. To clarify this, the AGM region was sub-dissected into four parts along the rostro-caudal axis as outlined in Figure S2. Part 1 was cut above the urogenital ridges (UGRs) and included the aortic bifurcation. Part 2 was cut from above the vitelline artery/superior mesenteric artery (VA/SMA) to the rostral border of the UGRs (the superior mesenteric artery is a derivative of the vitelline artery and supplies the mid gut; Schoenwolf et al., 2014). Part 3 was cut above the umbilical artery (UA) entry points and included the VA/SMA, and part 4 included the UA entry points and aorta below. Each sub-section of the AGM region was cultured as an explant in the presence of SCF 100 ng/mL and after 7 days transplanted into irradiated adult NSG recipients.

In four of these five independent "mapping" experiments, HSCs could be detected in AGM regions after culture (Table 1, Experiments E–I). In all successful experiments, HSCs were found in parts 2 and 3, i.e., around and above the VA/SMA limited by the rostral border of the UGRs. In one of these experiments, an additional HSC was also found in part 4, around the UA entry points. Interestingly, it seems that we had a slight expansion of HSC numbers in these experiments. Three of four cultured CS16–17 AGM regions contained two or three HSCs, which is higher than expected in fresh AGM regions (Ivanovs et al., 2011). Similarly, we have been able to detect the appearance of a definitive HSC after transplantation of part 2 of a CS13 embryo, which has not previously been detectable in the AGM region at this stage.

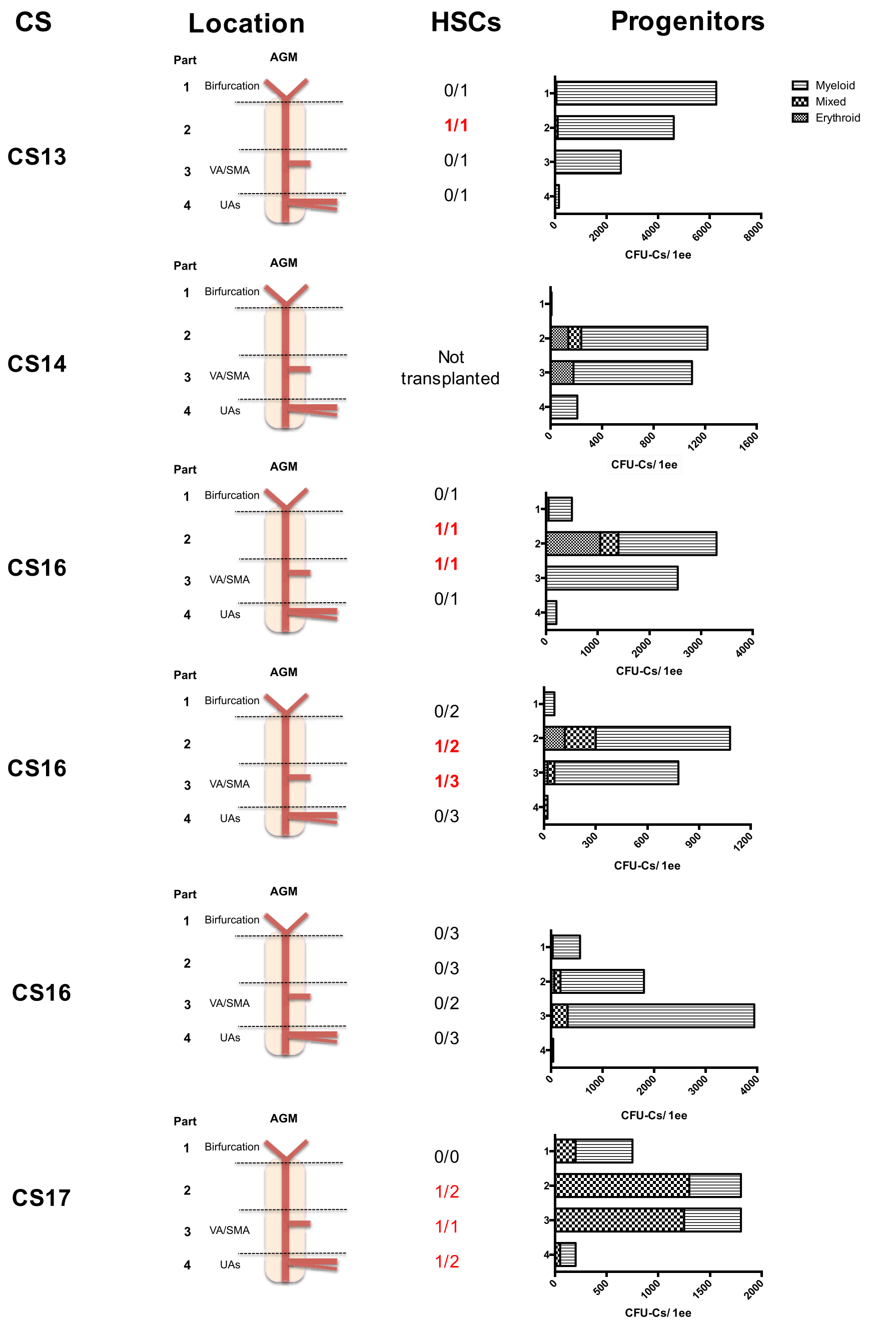

Figure 3. Relative Spatial Distribution of CFU-Cs and HSCs from the Human AGM after Culture

Mapping experiments demonstrating the location of HSCs and CFU-Cs in each of four parts of the AGM (see Figure S2) after 7 days of explant culture with addition of SCF 100 ng/mL are shown. Numbers and types of CFU-Cs are shown along with the Carnegie stage (CS) of each independent experiment. The corresponding number of mice with long-term multilineage engraftment relative to the number of mice injected is shown for each part of the AGM. The repopulated mice correspond to those shown in Tables 1, 2, and 3. ee, embryo equivalent; UAs, umbilical arteries; VA/SMA, vitelline artery/superior mesenteric artery. See also Figure S2.

To establish the relative location of CFU-C production in the AGM region, a small aliquot, no greater than 0.1 embryo equivalent (ee), was taken from the single-cell suspension made from embryonic tissues 7 days after culture (the rest was used for transplantations into NSG mice as described above; Table 1). The aliquot was used for the hematopoietic CFU-C assay. Results from six independent experiments from CS13–17 are shown in Figure 3. Parts 2 and 3 contained the highest numbers of CFU-Cs in all six experiments (range, 780–4,600), except for the CS13 experiment where part 1 was found to have the most CFU-Cs. HSCs were also consistently present in parts 2 and 3 (Figure 3 and Table 1). In all six experiments, the lowest CFU-C numbers ($\leq$210 per 1 embryo equivalent [ee]) were detected around the UA entry points. Thus, the location of the highest CFU-C numbers correlated with the positions in which HSCs were detected. In all experiments, except CS13 analysis, mixed colonies were

found after culture in addition to myeloid and erythroid colonies (Figure 3).

Explant Culture Maintains HSCs with Long-Term Multilineage *In Vivo* Repopulating Capacity

In NSG mice repopulated with cultured human AGM, engraftment could first be detected in the peripheral blood of mice at around 8 weeks (0.14% ± 0.1%), and in most there was a notable rise in engraftment level at 4–5 months (Figure 4A). Levels of engraftment ranged from 1.6% (12 weeks) to 49.7% (17 weeks) in the bone marrow, 0.1% (12 weeks) to 10% (17 weeks) in the blood, and 0.4% (12 weeks) to 20.8% (20 weeks) in the spleen (Table 2). The one NSG recipient successfully engrafted with cultured embryonic liver had relatively higher engraftment at 21 weeks compared with those transplanted with the human AGM with 22% in blood, 23.4% in bone marrow, and 23.9% in the spleen (Table 2). As expected, across all samples, human multilineage hematopoietic repopulation generally displayed a B lymphoid bias in engrafted NSG mice (Ivanovs et al., 2011; Shultz et al., 2005; Theocharides et al., 2016). Human $CD19^{+}$ B cells were the major human leukocyte population in the peripheral blood (55% ± 18%) and spleen (58% ± 24%). However, in the bone marrow, the major human leukocyte population was more variable, in some mice being B lymphoid-biased (9/13 mice; mean, 52% ± 24%) and in others myeloid-biased (4/13 mice; mean, 37% ± 22%) (Table 3, Figures 4B and S4). Human $CD33^{+}CD66b^{+}$ granulocytes and $CD33^{+}CD14^{+}$ macrophages were also detected in blood, bone marrow, and spleen of engrafted NSG mice (Table 3, Figures 4B, 4C, and S4). The level of human $CD3^{+}$ T cells and $CD94^{+}$ NK cells was particularly variable, as had been seen with the transplantation of fresh AGM into NSG mice (Ivanovs et al., 2011). Human immunophenotypic HSCs

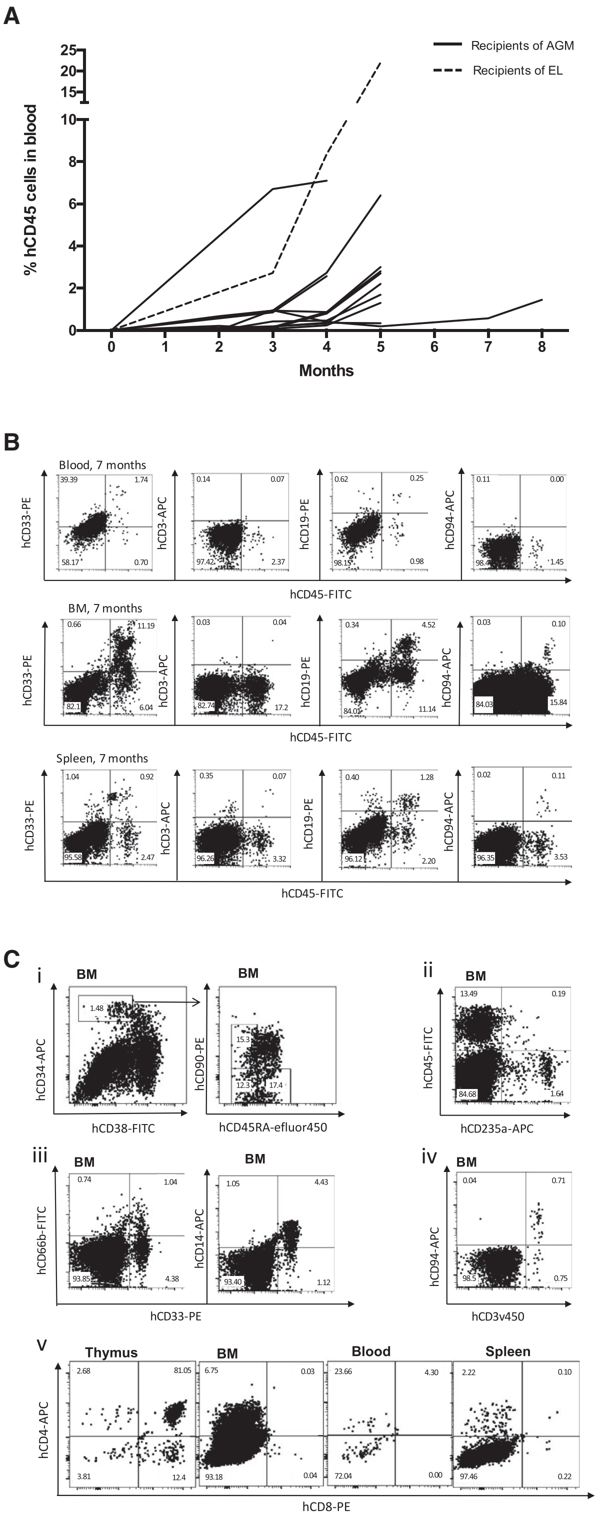

Figure 4. Explant Culture Maintains HSCs with Long-Term Multilineage Repopulating Capacity *In Vivo*

(A) Kinetics of human hematopoietic repopulation in the peripheral blood for 14 NSG mice transplanted with cultured human AGM (solid lines) or embryonic liver (EL) (dotted line).

(B) Representative flow cytometry plots of human myeloid ($CD45^{+}CD33^{+}$), T ($CD45^{+}CD3^{+}$), B ($CD45^{+}CD19^{+}$), NK ($CD45^{+}CD94^{+}$) cells in the peripheral blood, bone marrow, and spleen of an NSG mouse transplanted with 0.3 ee of a cultured CS16 human AGM region (part 2), culled for analysis at 7 months.

(C) Representative flow cytometry plots of HSCs ($CD34^{+}CD38^{-}CD90^{+}CD45RA^{-}$), multipotent progenitors (MPPs) ($CD34^{+}CD38^{-}CD90^{-}CD45RA^{-}$), and multi-lymphoid progenitors (MLPs) ($CD34^{+}CD38^{-}CD90^{-}CD45RA^{+}$) (i), erythroid cells (ii), myeloid cells (iii), NK/NKT cells (iv), and T cells (v) in the bone marrow, blood, spleen, and thymus of NSG mice. (i), (ii), and (v) are from a primary recipient mouse injected with 0.96 ee of AGM part 3 from a CS16 embryo cultured for 7 days, culled for analysis at 4 months. (iii) and (iv) are taken from a primary recipient mouse injected with 0.28 ee AGM posterior half, from a CS17 embryo cultured for 7 days, culled for analysis at 5 months.

All plots are gated on 7AAD singlets. Flow cytometry controls are shown in Figure S4. BM, bone marrow.

Table 2. Level of Repopulation in Mice Engrafted by Cultured Human Embryonic HSCs

Experiment No. (CS)	AGM Region	Time after Transplantation (Weeks)	% Human CD45$^+$ Cells Blood	Bone Marrow	Spleen
B (16)	Anterior	17	10.0	49.7	7.7
C (17)	Posterior	20	2.2	16.8	20.8
		20	1.3	3.3	7.5
		20	6.4	32	10.5
D (17)	Whole	12	0.1	1.6	0.4
E (13)	Part 2	19	0.3	41.1	8.3
F (16)	Part 2	16	0.9	15.7	0.6
	Part 3	16	2.6	27.2	3.1
G (16)	Part 2	27	1.6	15.8	3.3
	Part 3	33	1.5	10.8	5.6
I (17)	Part 2	21	3.0	22.4	6.8
	Part 3	21	2.7	12.1	2.8
	Part 4	21	2.8	10.8	2.9
	Liver	21	22.0	23.4	23.9

The level of repopulation of engrafted mice only is shown and corresponds to the engrafted mice indicated in bold in Table 1. (For example, Part 1 never gave engraftment, hence is not seen in this table). Parts 1–4 are described in Figure S2 and experiments correspond to those in Tables 1 and 3. The NSG mouse engrafted with embryonic liver is included for completeness. CS, Carnegie stage.

($CD34^+CD38^-CD90^+CD45RA^-$), multipotent progenitors (MPPs) ($CD34^+CD38^-CD90^-CD45RA^-$), and multilymphoid progenitors (MLPs) ($CD34^+CD38^-CD90^-$ $CD45RA^+$) could be identified in the recipient bone marrow (Figure 4C) (Doulatov et al., 2010; Majeti et al., 2007), constituting 12% ± 4%, 13% ± 4%, 23% ± 7% of $CD34^+CD38^-$ cells, respectively. Comparing these proportions with human bone marrow (BM) and cord blood (Doulatov et al., 2010; Majeti et al., 2007), MLPs were the dominant population of hematopoietic progenitors in the recipient BM rather than MPPs.

DISCUSSION

The AGM region is the major site of HSC development in mammals. The first HSCs emerge in low numbers (as single cells), both in the mouse and human dorsal aorta (Ivanovs et al., 2011; Kumaravelu et al., 2002; Müller et al., 1994). It had previously been shown that the first HSCs emerge from the ventral side of the dorsal aorta at CS14–17 in the human embryo (Ivanovs et al., 2014). However, their more precise location along the rostral-caudal axis and relationship to CFU-Cs remains unexplored. In addition, the developing human HSC hierarchy has yet to be established. While an *ex vivo* culture system has facilitated recapitulation of HSC development in the mouse and been pivotal in our understanding of the mouse developmental HSC hierarchy, this system has not yet been translated into human studies (Rybtsov et al., 2011, 2014; Souilhol et al., 2016; Taoudi et al., 2008). We therefore sought to advance understanding of early human hematopoietic development by temporal and spatial characterization of CFU-Cs and HSCs through adaption of the murine *ex vivo* culture system for the human AGM region. A previous study described the distribution of CFU-Cs in the extra-hepatic embryo but did not specifically describe CFU-Cs in the AGM (Huyhn et al., 1995).

We observed significant numbers of CFU-Cs initially in the yolk sac from CS12. Meanwhile, the AGM region was only gradually accumulating CFU-C numbers in line with the role of the yolk sac in the colonization of the AGM region established for the mouse (McGrath et al., 2015; Palis et al., 1999). Interestingly, we observed a sharp rise in CFU-C content in the liver beginning from CS15, consistent with previous studies (Huyhn et al., 1995; Tavian et al., 1996, 1999). It is not currently clear whether it is related to changes in CFU-C properties or critical development of the liver stroma. Notably, we observed the appearance of immature CFU-MIX in the liver from CS16,

Table 3. Multilineage Hematopoietic Engraftment in Primary Recipient Mice

		Blood (%)				Bone Marrow (%)				Spleen (%)			
Experiment No. (CS)	Tissue	Myeloid	B	T	NK	Myeloid	B	T	NK	Myeloid	B	T	NK
B (16)	Anterior	28	30	40	0	8	90	0.3	0.2	3.9	88.2	0.7	0.1
C (17)	Posterior	29	69	0	0	42	47	1.5	0	32	62	5	0.5
		25	70	0	0	43	47	3.9	0.6	9	78	1.3	1.4
		7	45	45	2	34	52	8	4	30	8	60	1
D (17)	Whole	NA	NA	NA	NA	56	33	1	0	22	43	0	0
F (16)	Part 2	8	61	30	0	23	75	1.5	0.1	26	69	0.9	3
	Part 3	20	46	NA	0	30	69	0.7	0	20	NA	NA	NA
G (16)	Part 2	51	39	2	0	68	26	0.1	0.3	33	55	7	3
	Part 3	33	17	33	12	82	12	0.3	0.9	33	49	7	7
I (17)	Part 2	10	72	11	2.3	23	68	1.0	0.7	6	79	10	3.6
	Part 3	36	47	1.4	0.8	31	61	1	0.8	10	84	1.4	0.8
	Part 4	16	61	2.0	7	18	70	1.3	0.1	9	85	0.7	0
	Liver	12	22	48	17	70	18	12	2.6	18	38	43	10

Percentage of myeloid cells ($CD33^+$), B cells ($CD19^+$), T cells ($CD3^+$), and NK cells ($CD94^+$) in the human $CD45^+$ cell population in the peripheral blood, bone marrow, and spleen of repopulated primary recipient NSG mice is shown. Only repopulated mice are shown and correspond to engrafted mice indicated in Tables 1 and 2. The NSG mouse engrafted with embryonic liver is included for completeness. NA, not assessed.

but not in other tissues (without culture), consistent with the role of the liver in the development of definitive hematopoiesis. Future analysis will be needed to determine what proportion of CFU-Cs circulating during these stages are erythro-myeloid progenitors (McGrath et al., 2015) and which of them are multipotent ones with lymphoid potential. Based on analysis of the mouse model, we can assume that the human AGM region contains a number of immature precursors of definitive HSCs (Rybtsov et al., 2011, 2014; Taoudi et al., 2008). In line with that, AGM cultures start producing CFU-MIX, which are absent in the fresh tissue.

HSC precursors are unable to engraft when directly transplanted into an adult recipient, but can engraft when allowed to mature in culture to become HSCs. Our adaption of the murine *ex vivo* culture system failed to convincingly reveal HSC precursors but has hinted at their existence. We established that up to three HSCs can be detected after 7 days of explant culture of the human AGM region, which at least matches and potentially surpasses the one or two HSCs detected in the fresh AGM region (Ivanovs et al., 2011). Hence, definitive HSCs from the human AGM can, at least, be maintained *ex vivo*. In each of ten previous independent experiments, we detected one HSC per fresh AGM region (except one experiment when two HSCs were found) (Ivanovs et al., 2011). In the current experiments, we frequently detected at least two HSCs per AGM region, suggesting some limited maturation of HSC precursors in the explant culture system. Indeed, it seems unlikely that this slight effect was due to self-renewal of pre-existing definitive HSCs, since after culture, HSCs were generally present in separate AGM sub-sections. Although experiments directly comparing cultured and uncultured AGM would have helped to answer whether the culture system did indeed mature any precursors of HSCs, such experiments are not possible due to the scarcity of human embryonic tissue. Although the aggregate culture system could address this issue, we found no repopulation after aggregate culture of human AGM region.

HSCs derived from explant cultured human embryonic tissue provided long-term multilineage repopulation of NSG mice as was observed with fresh AGM region (Ivanovs et al., 2011). Variability in the distribution of lineages engrafted was detected, in line with our previously reported transplantation of AGM region cells directly into NSG mice (Ivanovs et al., 2011). This may reflect individual differences in the repopulating capacity of NSG mice or the lineage bias of individual HSCs transplanted. Proportions of progenitor populations in the BM of repopulated mice differed from those previously reported for fresh BM and cord blood (Doulatov et al., 2010; Majeti et al., 2007). Again, further work would be required to investigate

whether this is due to unique properties of human embryonic HSCs, the repopulating capacity of NSG mice, or both. It was noted that the absolute levels of human engraftment reported here were slightly lower than those previously reported for human AGM directly transplanted into NSG mice without being cultured (Ivanovs et al., 2011). Given the time between the studies, this may reflect many variables, including the individual NSG mice. However, it could reflect a real reduction in the repopulating capacity of the HSCs after culture.

In terms of spatial distribution, this study has shown that HSCs are chiefly located around and above the vitelline artery entry point to the rostral end of UGRs. HSCs in the E11 mouse AGM region were also found to be enriched in the middle portion of the aorta, near the junction of the dorsal aorta and vitelline artery (Mascarenhas et al., 2009). Notably, these AGM portions possess the capacity to produce large numbers of CFU-Cs in our experiments. It is also interesting that this area of the human AGM region is known to be enriched for IAHCs (Tavian et al., 1996, 1999). Nonetheless, correlation between IAHCs and HSC activity is not direct; by CS17, IAHCs largely disappear, while HSCs are still detectable (Ivanovs et al., 2011; Tavian et al., 1999). Here, we show that CFU-C productivity in the AGM region increased in a linear manner throughout CS12–17, while the IAHCs decline by CS17. This may be due to migration of progenitors from the yolk sac via the vitelline artery and colonization in the AGM region. However, further work would be required to examine this hypothesis.

This work poses the question of why this *ex vivo* culture system does not facilitate the large-scale maturation and expansion of human HSCs seen for mouse HSCs, given the robustness of the mouse AGM culture system. The most likely explanation is that the culture system has not been optimized. Given the significantly longer timescale of human development (Ivanovs et al., 2017), HSC maturation may require more than the 7 days that we were testing in these experiments. Additionally, here we chose factors based on mouse *ex vivo* culture studies (Robin et al., 2006; Taoudi et al., 2008). In spite of significant conservation of hematopoietic development across vertebrate species, there might be differences in requirement of cytokines between mouse and human.

In summary, we established that human AGM-derived definitive HSCs can be maintained and on rare occasions mature from HSC precursors in culture. This work enhanced our understanding of the spatial development of HSCs, localizing them predominantly to the portion of the AGM region around and above the vitelline artery, which shows high CFU-C productivity, and which are enriched for intra-aortic clusters. Our study highlights important differences in the biology of human and murine embryonic hematopoietic development, which are yet to be unraveled.

We believe the search for conditions to support human HSC development in human AGM culture should continue, as this would greatly assist the derivation of transgene-free clinically applicable HSCs from human embryonic/induced pluripotent stem cells.

EXPERIMENTAL PROCEDURES

Processing of Human Embryonic Tissue

Human embryos were obtained immediately after elective termination of pregnancy either from the Royal Infirmary of Edinburgh (RAA/FT study, approved by the Lothian Research Ethics Committee, Reference: 08/S1101/1) or from the MRC-Wellcome Trust Human Developmental Biology Resource (HDBR), Institute of Genetic Medicine, Newcastle. All patients donating tissue had given informed written consent for the use of human embryonic tissues in research prior to tissues being obtained and anonymized. The developmental stage of human embryos was determined by the CS system (Ivanovs et al., 2013; O'Rahilly and Muller, 1987).

Dissections were performed under a low power stereomicroscope (Leica MZ8) in sterile dissection buffer (Dulbecco's phosphate buffered saline [PBS] with Mg^{2+} and Ca^{2+} ions [Sigma-Aldrich] containing 7% fetal calf serum [FCS] [Thermo Fisher Scientific or APS], 100 IU/mL penicillin and 100 μg/mL streptomycin [Invitrogen]). Where possible, the AGM region, yolk sac, umbilical cord, vitelline vessels, and liver were obtained from each embryo. However, due to damage to the embryo prior to obtaining them, it was not always possible to harvest all tissues. Further sub-dissection of the AGM region is described in the Results. Images of human tissue were taken using a Leica MZ FLIII with DFC7000T camera (Leica). Embryonic tissues were dissociated to single-cell suspensions in 5 mL polystyrene tubes (BD Falcon). For this, 800 μL of dissection buffer was used with 100 μL of 10 mg/mL collagenase-dispase (Roche) and 100 μL of 1.2 mg/mL DNase I (Roche) and incubated at 37°C for 40 min with gentle shaking in a water bath. The dissociation was stopped by adding 4 mL of dissociation buffer (Dulbecco's PBS without Mg^{2+} and Ca^{2+} [Sigma-Aldrich] containing 7% FCS and 100 IU/mL penicillin and 100 μg/mL streptomycin), centrifugation at 350 × *g* for 5 min at 4°C and resuspending in flow cytometry buffer (Dulbecco's PBS without Mg^{2+} and Ca^{2+} containing 2% FCS with or without 100 IU/mL penicillin and 100 μg/mL streptomycin) in a suitable volume for further analysis or culture. The number of cells was quantified as embryo equivalents. "Independent" experiments refers to those performed with different human embryos.

Human Embryo Culture System

Human embryonic tissues were cultured both as explants and aggregates in Costar tissue culture 6-well plates in embryo culture medium (Iscove's modified Dulbecco's medium [IMDM; Gibco], 20% pre-selected heat-inactivated Hyclone FCS [Thermo Fisher Scientific], L-glutamine [4 mM, Invitrogen], 100 IU/mL penicillin and 100 μg/mL streptomycin). The culture was performed at the liquid-gas interface on 0.8-μm nitrocellulose filters (Millipore)

(pre-washed in culture medium) at 37°C in 5% CO_2 for 7 days unless otherwise stated. The medium was changed at 24 h then left for a further 6 days. For explants, each embryonic tissue was placed on its own membrane immediately after dissection. For the aggregate system, both self-reaggregates and co-aggregates with OP9s were made. In both cases, a single-cell suspension of the tissue was first obtained as described above. OP9 cells were maintained in IMDM (Invitrogen), 20% FCS supplemented with L-glutamine (4 mM), penicillin/streptomycin (50 U/mL) and passaged every 4–5 days. They had previously been maintained in the Medvinsky group. Where co-aggregates were being made, OP9 stromal cells were first obtained by trypsinization and mixed with a single-cell suspension of embryonic tissue (1×10^5 OP9s + 1×10^5 human embryonic cells/aggregate). For self-reaggregates, 1×10^5 embryonic cells were used per aggregate. Two to three aggregates were placed on each membrane. Reaggregates or co-aggregates were formed by centrifugation of cell suspensions at $440 \times g$ for 12 min in 200-μL pipette tips sealed with parafilm to form a pellet (Taoudi et al., 2008). Experimental variables for the culture medium included addition of human SCF 100–300 ng/mL (catalog no. 300-07), IL3 100 ng/mL (catalog no. 200-03), FLT3LG 100 ng/mL (catalog no. AF-300-19) (all Peprotech), TGF-β inhibitor (SB 431542) 5 μM (Tocris, catalog no. 1614), ROCK inhibitor Y-27632 10 μM (Sigma-Aldrich, catalog no. SCM075). Details are described in the Results section. Both explants and aggregates were harvested and dissociated with collagenase/dispase as outlined for fresh embryonic tissues and used for further analysis.

In Vivo Long-Term Repopulation Assay

NOD.Cg-Prkdcscid Il2rgtm1Wjl (NSG) mice were used as recipients of human cells. All experiments with animals were performed under a project license granted by the Home Office (UK), University of Edinburgh Ethical Review Committee, and conducted in accordance with local guidelines. Animals were bred and housed within the University of Edinburgh under the regulations of the Animals Scientific Procedures Act, UK, 1986. The NSG mice were housed in pathogen-free conditions in individually ventilated cages or isolators. They received autoclaved drinking water, autoclaved bedding, and γ-irradiated chow diet. For the first 4 weeks after irradiation, their autoclaved drinking water was supplemented with 0.1 mg/mL of enrofloxacin (10% Baytril solution from Bayer). All mice were exposed to a constant cycle of 14 h of light/10 h of dark. Up to 6 h prior to transplantation with human cells, 6- to 8-week-old NSG mice received a sublethal dose of total body irradiation (3.3 Gy at a rate of 0.75 Gy/min from a ^{137}Cs source [GSR D1 γ-irradiator, Gamma-Service Medical]). Animals were transplanted with cells intravenously via the lateral tail vein in up to 200 μL using a 26-gauge syringe needle (BD Microlance), according to the procedures described in the project license and the regulations of the Animals Scientific Procedures Act, UK, 1986. Where human embryonic tissue was used, the quantity of human embryonic tissue transplanted is expressed in embryo equivalents, which is the number of cells present in a whole or specified part of an embryo of that stage.

As a positive control for selected experiments, 4×10^4 UCB $CD34^+$ cells were injected intravenously. Human UCB samples were obtained from full-term umbilical cord after elective cesarean sections from the Edinburgh Reproductive Tissue Biobank. This study was approved by the Lothian Research Ethics Committee (reference, 16/SS/0220). All patients donating tissue had given informed written consent for the use of human tissues in research prior to tissues being obtained and anonymized. Erythrocytes were lysed with BD Pharm Lyse Buffer (BD Biosciences). $CD34^+$ cells were sorted by magnetic cell separation using CD34 microbeads, a QuadroMACS separator, and LS columns (MACS Miltenyi Biotec) according to the manufacturer's instructions. After selection, cells were stored at −150°C.

To detect long-term hematopoietic repopulation, NSG recipient mice were bled every 1–2 months starting from 6–8 weeks after transplantation. Their tail vein was nicked and 2–3 drops collected into flow cytometry buffer supplemented with 5 mM of EDTA (VWR International). Red cells were lysed using BD Pharm Lyse Buffer (BD Biosciences) diluted 1:10 with water, and flow cytometry was performed to assess for human engraftment with anti-human CD45 antibody. Experiments were ended at 3–8 months after transplantation. All recipient mice were killed by dislocation of the neck according to Schedule 1 of the Animal (Scientific Procedures) Act 1986, and their BM (long bones), spleen, and thymus were harvested. Spleen and thymus were gently mashed in a small volume of buffer through a 40 μm EASYstrainer (Greiner Bio-One). The BM was flushed out from the bones, and a single-cell suspension was prepared by gentle pipetting. Cell suspensions of BM, thymus, and spleen were analyzed by flow cytometry to detect long-term multilineage engraftment. Engraftment with human hematopoietic tissues was defined as >1% human $CD45^+$ cells in the BM with human cells also detectable in the blood and spleen by flow cytometry. Engraftment data are stated in the text as mean ± standard deviation.

Human Colony-Forming Cell Assay

The colony-forming cell assay (CFU-C assay) was used to detect hematopoietic progenitor numbers. Cells were plated in at least duplicate for all independent experiments, and the mean score was used for analysis. All graphs show means of independent experiments, i.e., biological not technical variance, rounded to the nearest 5 CFU-Cs. To detect CFU-C number in dissociated human embryonic tissue, 0.1–0.005 ee of cells were plated into 1.5 mL of MethoCult H4034 Optimum Medium (STEMCELL Technologies) in 30 mm untreated tissue culture dishes. Cells were incubated at 37°C in 5% CO_2. Hematopoietic colonies were counted, scored, and photographed after 10–14 days.

Flow Cytometry

Antibody staining was performed in 5 mL polystyrene tubes (BD Falcon) or in U-bottomed 96-well plates (Sterilin). For flow cytometry analysis, the following mouse anti-human monoclonal antibodies were used: CD3-APC (clone SK7, eBioscience), CD4-APC (clone RPA-T4, BD Biosciences), CD8-PE (clone RPA-T8, BD Biosciences), CD14-APC (clone M5E2, BD Biosciences), CD19-PE (clone HIB19, eBioscience), CD33-PE (clone WM53, BD Biosciences), CD34-APC (clone 8G12, BD Biosciences), CD38-FITC (clone HIT2, BD Biosciences), CD45-FITC (clone H130, eBioscience), CD45RA-efluor450 (clone HI100, eBioscience),

CD66b-FITC (clone G10F5, BD Biosciences), CD90-PE (clone 5E10, BD Biosciences), CD94-APC (clone HP-3D9, BD Biosciences), CD235a-APC (clone GA-R2, BD Biosciences), and the rat anti-mouse monoclonal antibody, CD45-APC (clone 30-F11, eBioscience). The concentration used was as per the manufacturer's instructions or determined by in-house titration. Flow cytometry buffer was used for staining and washes. To prevent unwanted binding of antibodies to Fc receptors, cells were resuspended in 25 μL of human FcR Blocking Reagent (Miltenyi Biotec) and anti-mouse CD16/32 purified monoclonal antibody (eBioscience) diluted 1:10 and 1:200, respectively, in flow cytometry buffer and incubated on ice for 10 min. Then 25 μL of antibody solution at the double concentration was added to the cells, making the final cell suspension volume 50 μL. Primary antibodies were incubated with cell suspensions for 30 min on ice in the dark prior to two washes. Cells were resuspended in flow cytometry buffer supplemented with 1.5 μg/mL of 7-amino-actinomycin (7-AAD) (eBioscience) for flow cytometry analysis. In parallel, control samples were stained with FMO (fluorescence minus one), in which all antibodies were added except for one. In addition, single stains for each antibody were prepared with cells, BD CompBeads Set Anti-Mouse Ig,κ (BD Biosciences) or UltraComp eBeads (eBioscience). Flow cytometry analysis was performed using a FACSCalibur (BD Bioscience) or BD LSR Fortessa analyzer (5 laser or 4 laser) (BD Bioscience). Compensation adjustments were made based on appropriate single stains, and gates were defined by FMO controls or isotype controls. Forward and side scatter profiles were used to assess cell size and granularity to gate cells of interest and exclude doublets. Live cells were gated according to low uptake of 7AAD. Flow cytometry data were analyzed with FlowJo v10.2 software (Tree Star).

SUPPLEMENTAL INFORMATION

Supplemental Information can be found online at https://doi.org/10.1016/j.stemcr.2019.03.003.

AUTHOR CONTRIBUTIONS

J.E. designed and performed experiments, prepared the figures, and wrote the manuscript. S.R., S.G.-K, A.I., and S.T. also designed and performed experiments. A.M. supervised the experimental design and interpretation and wrote the manuscript. R.A.A. facilitated access to human embryos through the RAA/FT study.

ACKNOWLEDGMENTS

We thank the patients for donating tissue and A. Saunderson, J. Creiger, and N. Forson for gaining consent from patients and collecting specimens. We thank J. Verth, C. Manson, and their staff for animal maintenance. This work was supported by the Wellcome Trust (grant 100468/Z/12/Z, J.E.) and the MRC (grant G0901577, A.M.).

Received: December 21, 2018
Revised: March 9, 2019
Accepted: March 11, 2019
Published: April 4, 2019

REFERENCES

Doulatov, S., Notta, F., Eppert, K., Nguyen, L.T., Ohashi, P.S., and Dick, J.E. (2010). Revised map of the human progenitor hierarchy shows the origin of macrophages and dendritic cells in early lymphoid development. Nat. Immunol. *11*, 585–593.

Huyhn, A., Dommergues, M., Izac, B., Croisille, L., Katz, A., Vainchenker, W., and Coulombel, L. (1995). Characterization of hematopoietic progenitors from human yolk sacs and embryos. Blood *86*, 4474–4485.

Ivanovs, A., Rybtsov, S., Welch, L., Anderson, R.A., Turner, M.L., and Medvinsky, A. (2011). Highly potent human hematopoietic stem cells first emerge in the intraembryonic aorta-gonad-mesonephros region. J. Exp. Med. *208*, 2417–2427.

Ivanovs, A., Rybtsov, S., Anderson, R.A., Turner, M.L., and Medvinsky, A. (2013). Postmenstrual gestational age should be used with care in studies of early human hematopoietic development. Blood *121*, 3051–3052.

Ivanovs, A., Rybtsov, S., Anderson, R.A., Turner, M.L., and Medvinsky, A. (2014). Identification of the niche and phenotype of the first human hematopoietic stem cells. Stem Cell Reports *2*, 449–456.

Ivanovs, A., Rybtsov, S., Ng, E.S., Stanley, E.G., Elefanty, A.G., and Medvinsky, A. (2017). Human haematopoietic stem cell development: from the embryo to the dish. Development *144*, 2323–2337.

Kumaravelu, P., Hook, L., Morrison, A.M., Ure, J., Zhao, S., Zuyev, S., Ansell, J., and Medvinsky, A. (2002). Quantitative developmental anatomy of definitive haematopoietic stem cells/long-term repopulating units (HSC/RUs): role of the aorta-gonad-mesonephros (AGM) region and the yolk sac in colonisation of the mouse embryonic liver. Development *129*, 4891–4899.

Majeti, R., Park, C.Y., and Weissman, I.L. (2007). Identification of a hierarchy of multipotent hematopoietic progenitors in human cord blood. Cell Stem Cell *1*, 635–645.

Mascarenhas, M.I., Parker, A., Dzierzak, E., and Ottersbach, K. (2009). Identification of novel regulators of hematopoietic stem cell development through refinement of stem cell localization and expression profiling. Blood *114*, 4645–4653.

McGrath, K.E., Frame, J.M., Fegan, K.H., Bowen, J.R., Conway, S.J., Catherman, S.C., Kingsley, P.D., Koniski, A.D., and Palis, J. (2015). Distinct sources of hematopoietic progenitors emerge before HSCs and provide functional blood cells in the mammalian embryo. Cell Rep. *11*, 1892–1904.

Medvinsky, A., and Dzierzak, E. (1996). Definitive hematopoiesis is autonomously initiated by the AGM region. Cell *86*, 897–906.

Müller, A.M., Medvinsky, A., Strouboulis, J., Grosveld, F., and Dzierzak, E. (1994). Development of hematopoietic stem cell activity in the mouse embryo. Immunity *1*, 291–301.

O'Rahilly, R., and Muller, F. (1987). Developmental Stages in Human Embryos (Carnegie Institute of Washington).

Palis, J., Robertson, S., Kennedy, M., Wall, C., and Keller, G. (1999). Development of erythroid and myeloid progenitors in the yolk sac and embryo proper of the mouse. Development *126*, 5073–5084.

Robin, C., Ottersbach, K., Durand, C., Peeters, M., Vanes, L., Tybulewicz, V., and Dzierzak, E. (2006). An unexpected role for IL-3 in

the embryonic development of hematopoietic stem cells. Dev. Cell *11*, 171–180.

Rybtsov, S., Sobiesiak, M., Taoudi, S., Souilhol, C., Senserrich, J., Liakhovitskaia, A., Ivanovs, A., Frampton, J., Zhao, S., and Medvinsky, A. (2011). Hierarchical organization and early hematopoietic specification of the developing HSC lineage in the AGM region. J. Exp. Med. *208*, 1305–1315.

Rybtsov, S., Batsivari, A., Bilotkach, K., Paruzina, D., Senserrich, J., Nerushev, O., and Medvinsky, A. (2014). Tracing the origin of the HSC hierarchy reveals an SCF-dependent, IL-3-independent CD43(-) embryonic precursor. Stem Cell Reports *3*, 489–501.

Rybtsov, S., Ivanovs, A., Zhao, S., and Medvinsky, A. (2016). Concealed expansion of immature precursors underpins acute burst of adult HSC activity in foetal liver. Development *143*, 1284–1289.

Schoenwolf, G.C., Bleyl, S.B., Brauer, P.R., and Francis-West, P.H. (2014). Larsen's Human Embryology, Fifth Edition (Churchill Livingstone).

Shultz, L.D., Lyons, B.L., Burzenski, L.M., Gott, B., Chen, X., Chaleff, S., Kotb, M., Gillies, S.D., King, M., Mangada, J., et al. (2005). Human lymphoid and myeloid cell development in NOD/LtSz-scid IL2R{gamma}null mice engrafted with mobilized human hemopoietic stem cells. J. Immunol. *174*, 6477–6489.

Souilhol, C., Gonneau, C., Lendinez, J.G., Batsivari, A., Rybtsov, S., Wilson, H., Morgado-Palacin, L., Hills, D., Taoudi, S., Antonchuk, J., et al. (2016). Inductive interactions mediated by interplay of asymmetric signalling underlie development of adult haematopoietic stem cells. Nat. Commun. *7*, 10784.

Taoudi, S., Gonneau, C., Moore, K., Sheridan, J.M., Blackburn, C.C., Taylor, E., and Medvinsky, A. (2008). Extensive hematopoietic stem cell generation in the AGM region via maturation of VE-cadherin^{+}CD45^{+} pre-definitive HSCs. Cell Stem Cell *3*, 99–108.

Tavian, M., Coulombel, L., Luton, D., Clemente, H.S., Dieterlen-Lièvre, F., and Péault, B. (1996). Aorta-associated CD34^{+} hematopoietic cells in the early human embryo. Blood *87*, 67–72.

Tavian, M., Hallais, M., and Péault, B. (1999). Emergence of intraembryonic hematopoietic precursors in the pre-liver human embryo. Development *126*, 793–803.

Tavian, M., Robin, C., Coulombel, L., and Péault, B. (2001). The human embryo, but not its yolk sac, generates lympho-myeloid stem cells: mapping multipotent hematopoietic cell fate in intraembryonic mesoderm. Immunity *15*, 487–495.

Taylor, E., Taoudi, S., and Medvinsky, A. (2010). Hematopoietic stem cell activity in the aorta-gonad-mesonephros region enhances after mid-day 11 of mouse development. Int. J. Dev. Biol. *54*, 1055–1060.

Theocharides, A.P.A., Rongvaux, A., Fritsch, K., Flavell, R.A., and Manz, M.G. (2016). Humanized hemato-lymphoid system mice. Haematologica *101*, 5–19.

Yokomizo, T., and Dzierzak, E. (2010). Three-dimensional cartography of hematopoietic clusters in the vasculature of whole mouse embryos. Development *137*, 3651–3661.

Stem Cell Reports, Volume *12*

Supplemental Information

Analysis of the Spatiotemporal Development of Hematopoietic Stem and Progenitor Cells in the Early Human Embryo

Jennifer Easterbrook, Stanislav Rybtsov, Sabrina Gordon-Keylock, Andrejs Ivanovs, Samir Taoudi, Richard A. Anderson, and Alexander Medvinsky

Table S1. Analysis of long-term repopulation by cultured human embryonic tissue (non-AGM). Relates to Table 1.

Experiment	CS	Tissue	Conditions	e.e./ mouse	no. engrafted/no. injected
**A**	15	UC	SCF, TGFβi, ROCKi	0.4	0/2
**B**	16	UC	SCF	0.25	0/4
**C**	17	EL	SCF, TGFβi, ROCKi	0.45	0+2 ^{A} /2
		UC		0.45	0/2
**E**	13	UC	SCF	0.5	0/2
		VA		0.5	0/2
		YS		0.5	0/2
**F**	16	UC	SCF	0.5	0/2
		VA		0.5	0/2
		YS		0.5	0/2
**H**	16	EL	SCF	0.4	0+1^{A}/1
**I**	17	EL	SCF	0.5	**1**/1
**J**	13	YS	SCF, FLT3L	0.3	0/3
**Long-term multilineage haematopoietic repopulation (HSCs)**<br>^{A}Low level (<1%) engraftment in bone marrow.					

The number of repopulated NSG mice relative to the number of mice injected and analysed are shown. The embryo equivalent (e.e.) received by each mouse refers to the e.e. of each *part* of the AGM region. Experiment letters correspond to experiments in Table 1. SCF is 100ng/ml unless stated as 300 (ng/ml), TGFβ inhibitor 5μM, ROCK inhibitor 10μM, FLT3L 100ng/ml. CS, Carnegie stage. TGFβi, TGFβ inhibitor. ROCKi, ROCK inhibitor. YS, yolk sac. UC, umbilical cord. VA, vitelline artery. EL, embryonic liver.

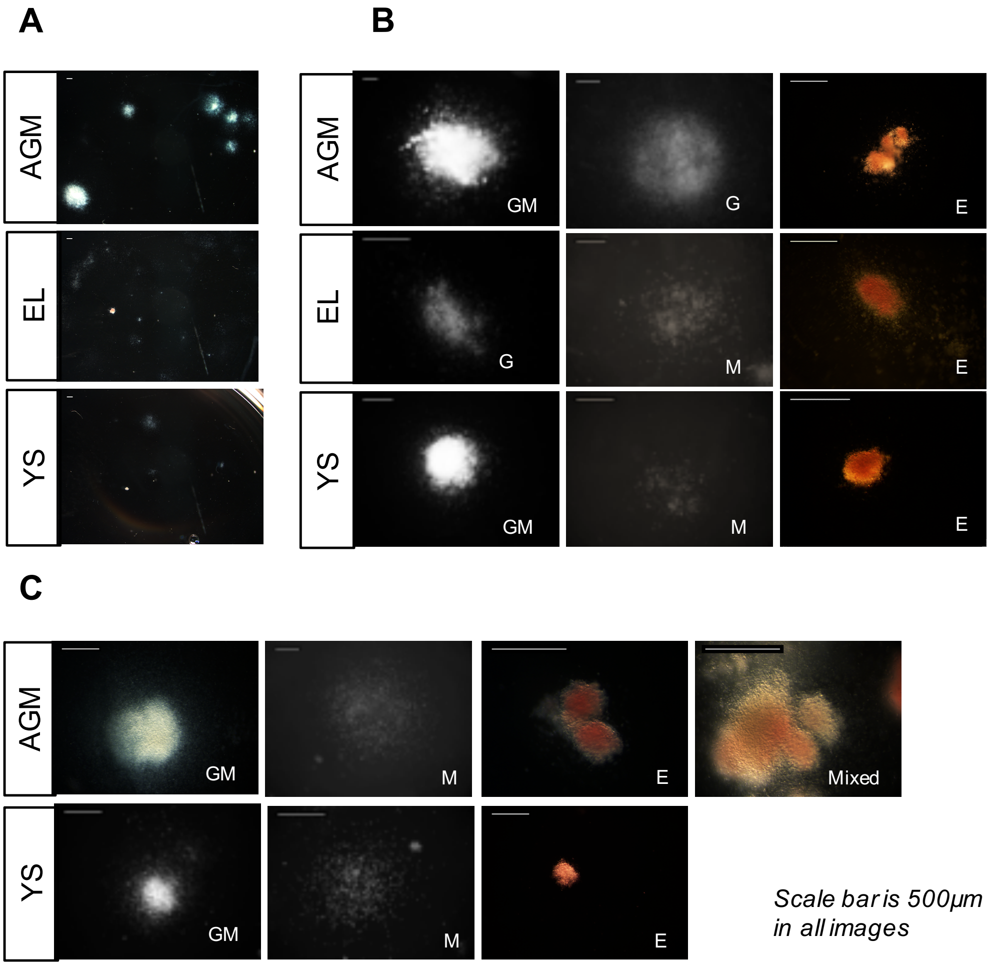

Figure S1. **CFU-Cs from human embryonic tissue before and after explant culture. Relates to Figure 1 and Figure 2B.**

Representative images of CFU-Cs from the AGM, embryonic liver (EL), and yolk sac (YS) before culture (A & B) and after 7 days of explant culture with SCF 100ng/ml (C). Images for all tissues were taken 10 days after cells were seeded in methylcellulose at appropriate embryo equivalents to allow accurate counting after culture. All scale bars represent 500µm.

A: CFU-Cs from a fresh (not cultured) CS16 embryo. These lower power images were all taken at the same magnification and show the different sizes of colonies from different tissues.

B: Example images of different colony types: Myeloid (granulocyte (G), macrophage (M), granulocyte/macrophage (GM)) and erythroid colonies are depicted for each tissue. No mixed colonies were produced from this embryo before explant culture.

C: CFU-Cs from a CS14 embryo after explant culture showing granulocyte/macrophage (GM), macrophage (M) and erythroid (E) colonies produced from both AGM and YS. Mixed colonies were produced from the cultured AGM tissue only.

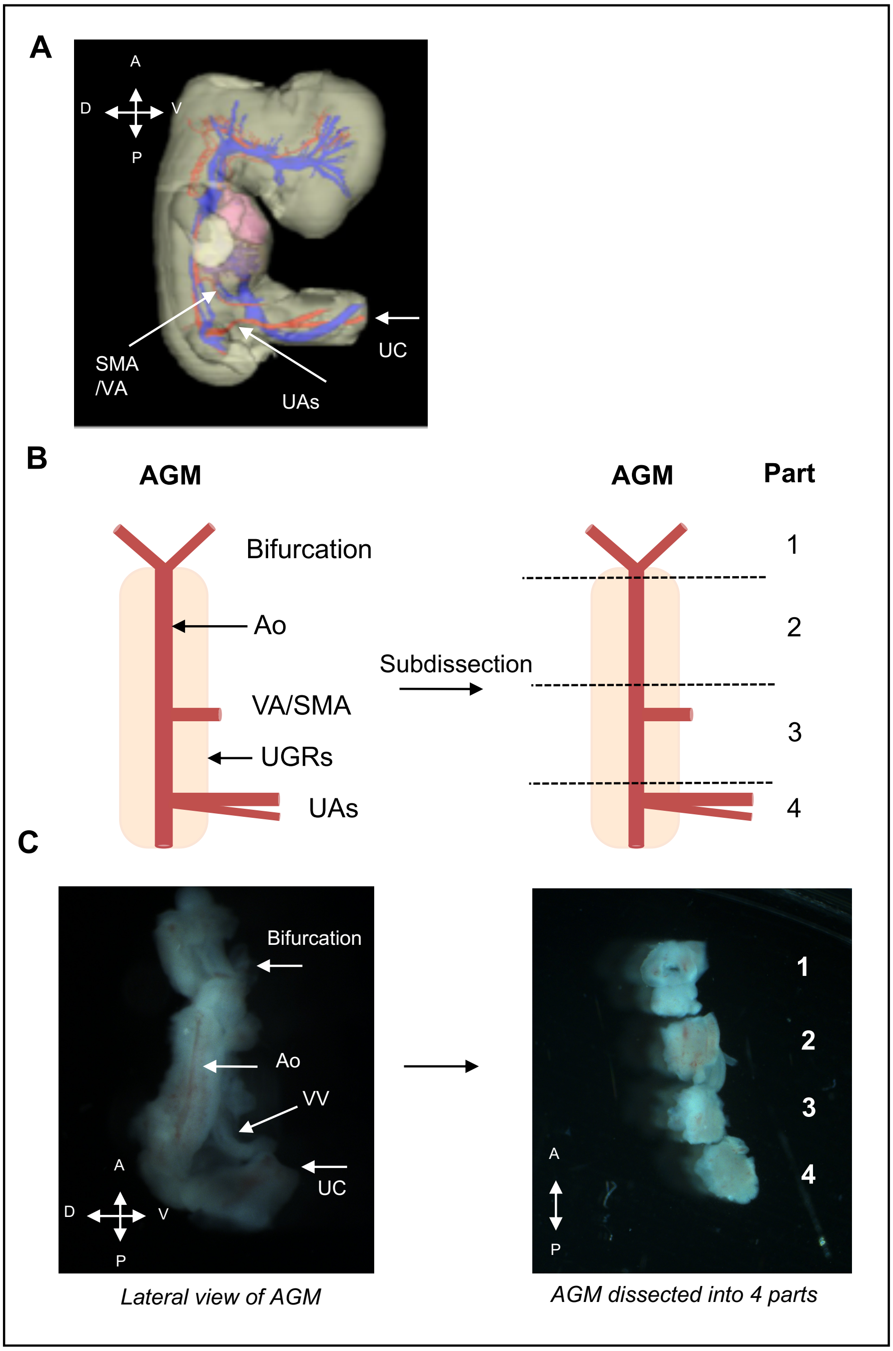

Figure S2. Sub-dissection of the human AGM along the rostro-caudal axis. Relates to Figure 3.
A: Circulatory system of a CS17 human embryo. Arteries (red) and veins (blue). The superior mesenteric artery (SMA), a derivative of the vitelline artery (VA) and umbilical arteries (UAs) within the umbilical cord (UC) are marked. Image adapted from *'The Virtual Human Embryo', hosted by EHD* (Gasser & Cork).
B: Schematic of sub-dissection to divide AGM into 4 regions along the rostro-caudal axis, performed for all CS13-17 embryos. Part 1: From the rostral part of the urogenital ridges (UGRs) including the aortic bifurcation
Part 2: From above the VA/ SMA to the rostral part of the UGRs
Part 3: From above the UAs entry points including the VA/ SMA
Part 4: Included the UAs entry points and the aorta below
C: Example of a CS17 embryo used in the experiments showing the lateral view of the AGM and the 4 parts after dissection.

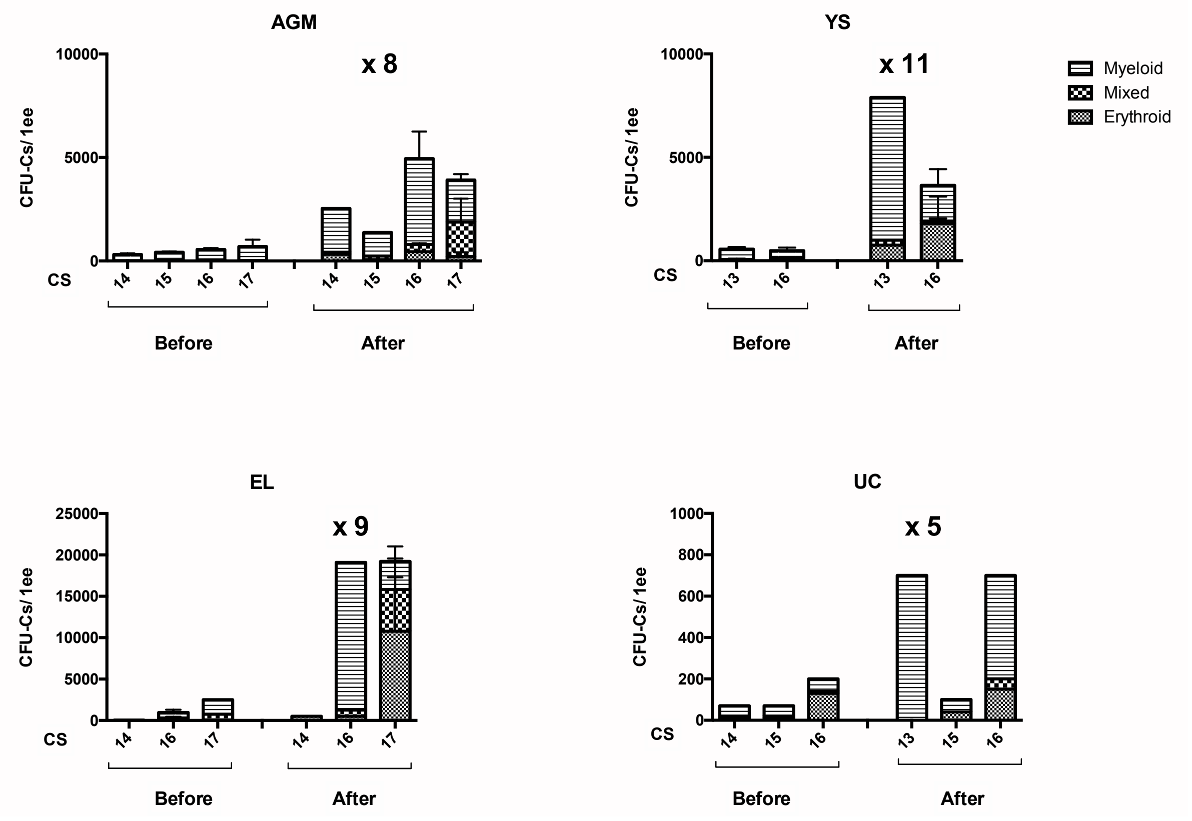

Figure S3. **Explant culture can expand committed haematopoietic progenitors from human embryonic tissue . Relates to Figure 2.**

Charts show the number and type (myeloid, mixed or erythroid) of CFU-Cs in the human AGM (A), yolk sac (YS) (B), embryonic liver (EL) (C), and umbilical cord (UC) (D) before and after explant culture for 7 days. Tissue was dissected and cultured at the air-liquid interface in standard embryo culture medium with addition of SCF 100ng/ml. The mean fold change in CFU-C numbers after culture is shown above the graph. Number of CFU-Cs before culture are the mean of separate experiments shown in Figure 1. n=1 except where error bars are given where n=2 and for AGM CS16 n=3. ee, embryo equivalent.

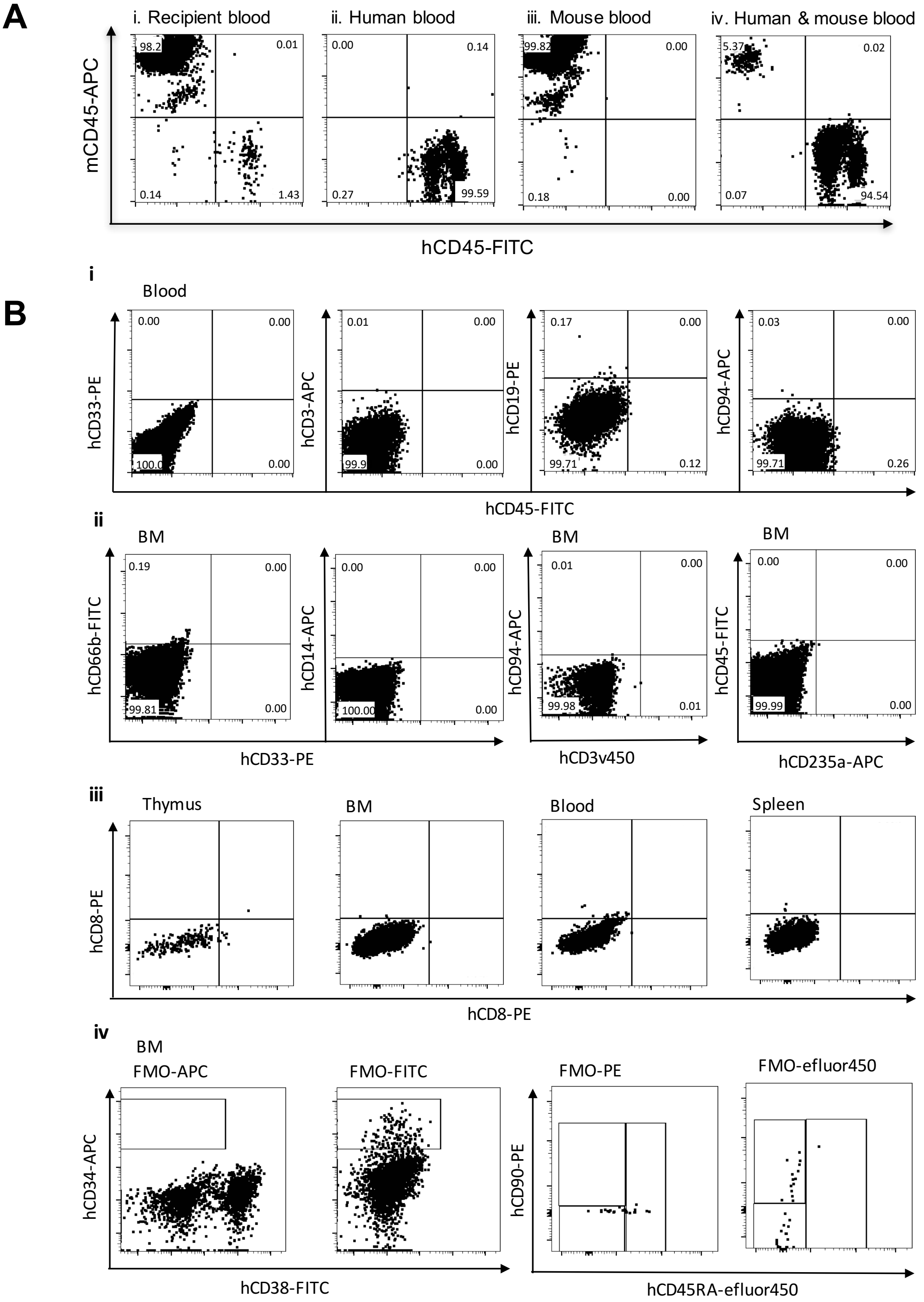

Figure S4. **Flow cytometry controls for multilineage analysis of haematopoietic repopulation. Relates to Figure 4.**

A: Representative plots showing analysis of peripheral blood of recipient NSG mice by flow cytometry. i. Engrafted recipient mouse blood from experiment G at 4 months, ii. Human peripheral blood (positive flow cytometry control), iii. Non-injected NSG mouse blood (negative control), iv. Human blood spiked with mouse blood, a control commonly used; plot iv was taken from a separate experiment from i-iii for illustration purposes.

B: Representative flow cytometry plots showing controls for gating human myeloid, T, B, NK cells (i), granulocytes, macrophages, NK/NKT cells and erythroid cells (ii), CD8/CD4 T cells (iii) and haematopoietic stem and progenitor cells (iv) in blood, bone marrow, spleen and thymus of primary recipient mice. Cells were generally gated using cells taken from non-engrafted primary recipient mice which had been sub-lethally irradiated & initially injected with PBS. This allowed gates to be drawn based on the autofluorescence of cells. Fluorescence minus one (FMO) controls were also used for each experiment and the gates drawn here were checked against each FMO control. Isotype controls were not used having carefully considered their advantages and disadvantages (Keeney *et al*, 1998; O'Gorman & Thomas, 1999).
